# Hypoxic Small Extracellular Vesicle Preconditioning of AC16 Cardiomyocytes Increase Caspase-3 and Caspase-8 Activity During Hypoxia

**DOI:** 10.3390/ijms262412123

**Published:** 2025-12-17

**Authors:** Øystein Røsand, Victoria Johansen, Gurdeep Marwarha, Morten A. Høydal

**Affiliations:** Department of Circulation and Medical Imaging, Faculty of Medicine and Health Sciences, Norwegian University of Science and Technology, 7491 Trondheim, Norway; oystein.rosand@ntnu.no (Ø.R.); victoria.johansen@ntnu.no (V.J.);

**Keywords:** small extracellular vesicles, myocardial ischemia, hypoxia, apoptosis

## Abstract

Small extracellular vesicles (sEVs) are increasingly recognized as crucial mediators of cell-cell communication. This study aims to determine the influence of sEV mediation on cardiomyocytes (CMs) under hypoxic conditions and identify the molecular modifications induced by hypoxia-derived sEVs (H-sEVs). Using a preconditional approach, we administered hypoxic AC16 CM-derived sEVs to recipient AC16 CMs before hypoxic stimulation. Molecular and biochemical analyses were performed to evaluate the biological effects of H-sEVs, e.g., cell viability assay, caspase assays, Western blotting, and quantitative sandwich ELISA. Our results showed a significant decrease in CM viability following hypoxic stimulation, as well as elevated caspase-3 and caspase-8 activity, and increased Bcl-2-associated X protein (BAX) translocation to the mitochondria followed by an increased release of Cytochrome C from mitochondria to cytosol. Preconditioning with H-sEVs further exacerbated caspase-3 and caspase-8 activation, which was explained by aggravated translocation of BAX to the mitochondria with consequences of cytochrome C release and increased apoptotic signaling. Our research indicates that sEVs secreted by hypoxic AC16 CMs negatively impact the biological properties of recipient CMs exposed to hypoxic stress. Thus, these findings widen our current understanding of sEV-mediated cellular communication during hypoxic events and provide insights into potential therapeutic targets for cardiac ischemic injury.

## 1. Introduction

In cardiovascular disease, ischemia plays a primary role in myocardial injury and cardiomyocyte cell death [1]. Ischemia is characterized by an insufficient blood supply to the myocardium which often results in tissue hypoxia [2]. The severity of hypoxia is dependent on various factors, such as the duration and magnitude of the hypoxic stress. Hypoxic stress may result in metabolic acidosis, an increase in reactive oxygen species (ROS), adenosine triphosphate (ATP) depletion, and calcium overload, ultimately causing myocardial damage if not addressed [3]. Moreover, hypoxia is known to activate both the intrinsic and extrinsic apoptotic pathways, exacerbating ischemic injury [4,5]. A well-studied mediator of cellular hypoxic adaptation is the hypoxia-inducible transcription factor 1α (HIF-1α), which plays a pivotal role in orchestrating oxygen-sensing and ischemic stress responses [6,7]. Under hypoxic conditions, stabilized HIF-1α can translocate into the nucleus, where it facilitates transcription of genes involved in angiogenesis, vascular remodeling, mitochondrial respiration, and cell survival [8,9,10]. Due to the pressing challenge of ischemia on human health, development of therapeutic strategies is vital to preserve myocardial function and reduce the burden of cardiovascular disease [11].

Over the past years, remote ischemic preconditioning (RIPC) has garnered considerable interest as a possible therapeutic strategy [12,13], as it has been shown to reduce ischemic myocardial damage during coronary artery bypass grafting [13] and cardiopulmonary bypass surgery [14]. The underlying protective effects involve multiple intracellular signaling pathways, many of which are regulated by HIF-1α [7,15]. Of particular significance is the reperfusion injury salvage kinase (RISK) pathway [16]. The RISK pathway elicits cardioprotection via activation of the pro-survival kinase protein kinase B (Akt), which exerts its protective effects by inhibiting pro-apoptotic proteins, e.g., the glycogen synthase kinase-3β (GSK3β) [17], Bcl-2-associated X protein (BAX) [18], and BCL2-homologous antagonist/killer (BAK) [19]. GSK3β activation promotes the intrinsic apoptotic pathway by activation of downstream pro-apoptotic mediators, e.g., BAX and BAK that may translocate to the mitochondria membrane and form mitochondrial apoptosis-induced channel (MAC), cytochrome C release, and activation of caspase-3 with consequences of apoptosis [20,21,22,23].

Small extracellular vesicles (sEVs) are gaining recognition as important mediators of cellular communication, influencing cardiac homeostasis and stimuli response, and have been shown to be involved in the cardioprotective effects of RIPC [24]. sEVs are a heterogenic population of naturally secreted particles carrying lipid-bilayer-encapsulated signaling molecules, such as proteins, lipids, DNA, coding and non-coding RNA, as well as metabolites [25,26]. They are primarily characterized by size (30–150 nm) and composition [27]. During ischemic events, sEVs are secreted by cardiomyocytes (CMs) as a response to the hypoxic stress, transporting signaling molecules, in a paracrine and endocrine fashion [28,29]. The cargo of sEVs varies depending on the cell’s condition at the time of secretion, influencing gene expression through transcriptional, post-transcriptional, and epigenetic regulation. Moreover, sEVs are increasingly recognized as potential biomarkers for clinical diagnosis and as therapeutic mediators of cardioprotection [30,31]. Recent findings indicate that plasma-derived sEVs from trans-aortic constriction mice can induce ventricular dysfunction and cardiomyocyte hypertrophy in healthy mice [32], suggesting that reducing pathological sEVs may be a potential strategy for treating cardiac disease, including ischemia [33]. Despite extensive research, it is therefore important to underscore that the complex signaling processes of sEVs during cardiac pathology, particularly ischemic events, are not yet fully understood. In this context, the present study was designed to explore the biological effects of hypoxia-derived sEVs (H-sEVs), with the aim of elucidating their potential contribution to cardioprotection. To achieve this, we employed a simulated preconditioning model using AC16 CMs to evaluate the impact of H-sEVs on apoptotic signaling and regulation of intracellular pathways responsible for CM stress responses.

## 2. Results

### 2.1. AC16 CMs Secrete sEVs into Condition Medium

**Figure 1:**
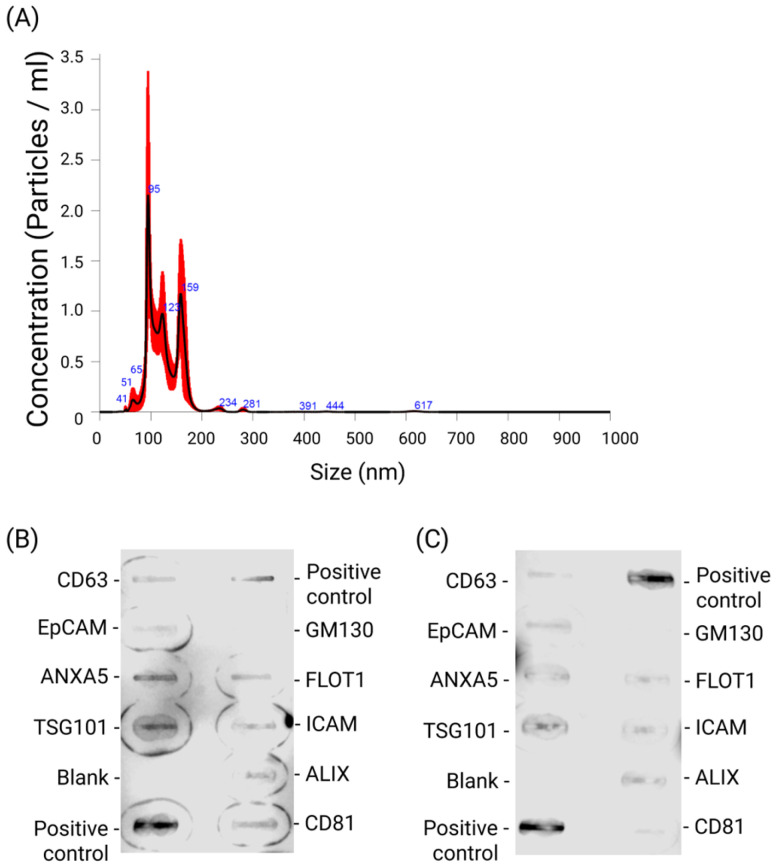
Isolated small extracellular vesicles (sEVs) were isolated from AC16 CM condition medium by the Total Exosome Isolation kit and analyzed using a Nanosight NS300. (**A**) NTA analysis displaying measured particle size and concentration distribution for AC16 CM-derived sEV. Particle sizes were captured five times for 60 s for every sample (*n* = 4). The mean diameter of sEVs was measured to be 126.7 nm (±4.4 nm), while the mean concentration of sEVs was measured to be 7.6 × 10^7^ sEV/mL (±1.76 × 10^7^ sEV/mL). To confirm the presence of established sEV protein markers, we utilized the Exo-Check™ Exosome Antibody Array. Membrane blots of pooled sample protein lysates (*n* = 6) from particles isolated from the (**B**) Hypoxic condition medium and (**C**) Normoxic condition medium demonstrated the detection of CD63, CD81, ALIX, FLOT1, ICAM1, EpCam, ANXA5, and TSG101. The cis-Golgi matrix protein GM130 was absent in all samples. NTA data were analyzed using the NanoSight Software NTA 3.2 Dev Build 3.2.16. Black line shows particle concentration while red error bars indicate ± SEM.

In order to investigate the biological impacts of sEVs secreted during hypoxia, H-sEVs were isolated from conditioned media of AC16 CMs and analyzed using a Nanosight NS300 (Figure 1A). NTA revealed a mean particle size of 126.7 nm (±4.4 nm). In addition, NTA measured sEV concentrations of 7.6 × 10^7^ sEV/mL (±1.76 × 10^7^ sEV/mL). The NTA analysis detected most particles within the 30–150 nm size range. This is in accordance with previous reports of sEV size range [34]. Furthermore, to confirm the presence of established sEV protein markers in the isolated particle samples, we employed the Exo-Check™ Exosome Antibody Array. Protein expression analysis of pooled particle samples (*n* = 6) isolated from both hypoxic and normoxic condition medium (Figure 1B,C) revealed the presence of CD63, CD81, ALIX, FLOT1, ICAM1, EpCam, ANXA5, and TSG101. Moreover, GM130, a cis-Golgi marker, was not detected in the samples. The combined results of NTA and protein validation confirm the effective isolation of sEV-sized particles from the normoxic and hypoxic AC16 condition medium.

### 2.2. Hypoxic Stimulation Increases HIF-1α Expression in AC16

**Figure 2:**
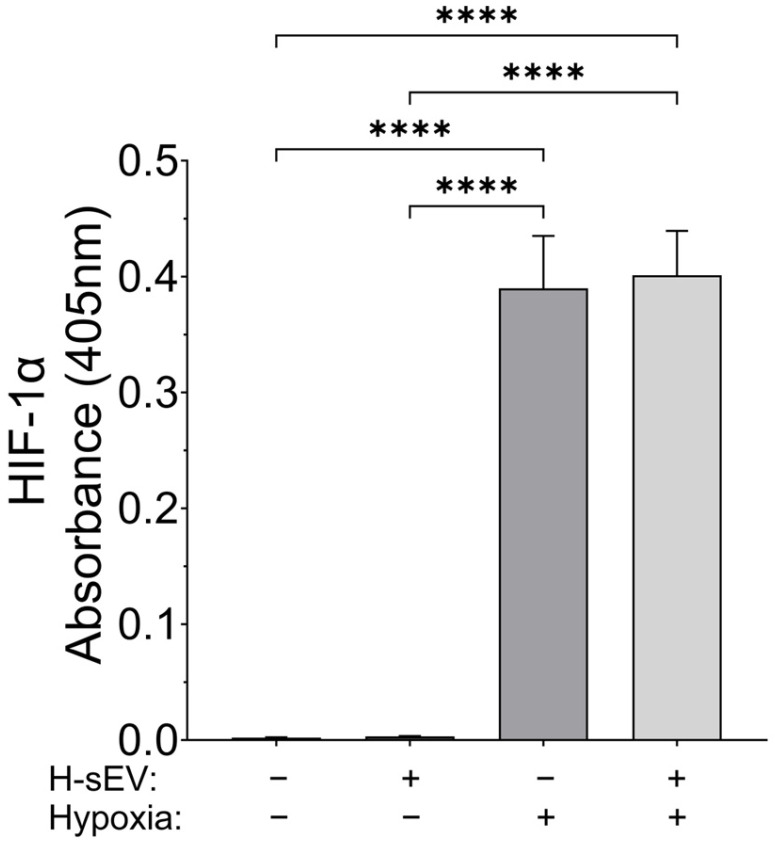
HIF-1α expression during hypoxia. Quantitative sandwich ELISA demonstrated a significant increase in the hypoxia-inducible factor 1α (HIF-1α) in AC16 cardiomyocytes (CMs) following 18 h of hypoxic stimulation. Preconditioning with H-sEVs for 24 h did not significantly alter HIF-1α expression compared with control cells under hypoxic conditions. Data were analyzed using one-way ANOVA multiple comparison analysis. Data is expressed as blank-corrected absorbances reported as mean ± SEM from three technical replicates for each of the four biological replicates belonging to each experimental group (*n* = 4). **** *p* ≤ 0.0001.

To verify the cellular response to the hypoxic stimulation in the AC16 CMs, we quantified HIF-1α levels following exposure to hypoxia. ELISA immunoassay (Figure 2) revealed a great increase in HIF-1α expression after 18 h of hypoxia in both preconditioned and untreated AC16 CMs compared with normoxic controls. Notably, no significant difference in HIF-1α levels was observed between H-sEV-preconditioned and control AC16 CMs under hypoxic conditions.

### 2.3. Hypoxia and H-sEVs Negatively Impact AC16

**Figure 3:**
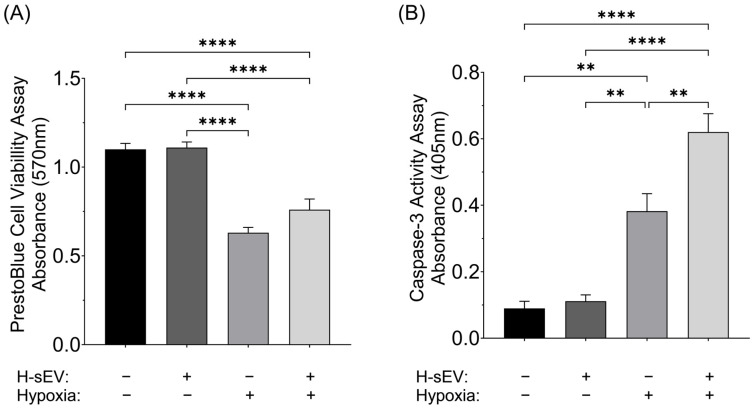
Cardiomyocyte viability and caspase-3 activation. (**A**) PrestoBlue cell viability assay shows that 18 h of hypoxia greatly decrease AC16 CM viability. Furthermore, H-sEV preconditioning of AC16 CMs did not significantly alter cell viability post 18 h of hypoxia. (**B**) Caspase-3 activity assay showed that 18 h of hypoxia increase caspase-3 activity in AC16 CMs. Furthermore, 24 h preconditioning with H-sEVs significantly increased caspase-3 activity (+63.2%) compared with control cells post hypoxia. Data was analyzed using one-way ANOVA multiple comparison analysis. Data from the PrestoBlue viability assay is expressed as mean of raw values ± SEM (*n* = 8). Data from the caspase-3 activity assay is expressed as mean of raw values ± SEM (*n* = 4). ** *p* ≤ 0.01; **** *p* ≤ 0.0001.

To investigate whether H-sEV preconditioning would affect cell viability during hypoxia, we performed a PrestoBlue cell viability assay (Figure 3A). Here we report that 18 h of hypoxia drastically decreased the cell viability of both H-sEV-preconditioned (−31.53%, *p* ≤ 0.0001) and control AC16 CMs (−47.72%, *p* ≤ 0.0001). Furthermore, H-sEV preconditioning did not significantly affect cell viability after 18 h of hypoxia. To further investigate the cellular responses to hypoxia and H-sEV preconditioning, we performed a caspase-3 activity assay (Figure 3B). In accordance with the cell viability assay, caspase-3 activity was greatly increased after 18 h of hypoxia for both preconditioned and non-treated AC16 CMs. However, interestingly, preconditioning with H-sEVs greatly increased caspase-3 activity compared with control cells (+63.2%, *p* ≤ 0.01) post hypoxia.

### 2.4. H-sEV Preconditioning Did Not Affect Akt, GSK3β, Bcl-2, or Bcl-XL Activity

**Figure 4:**
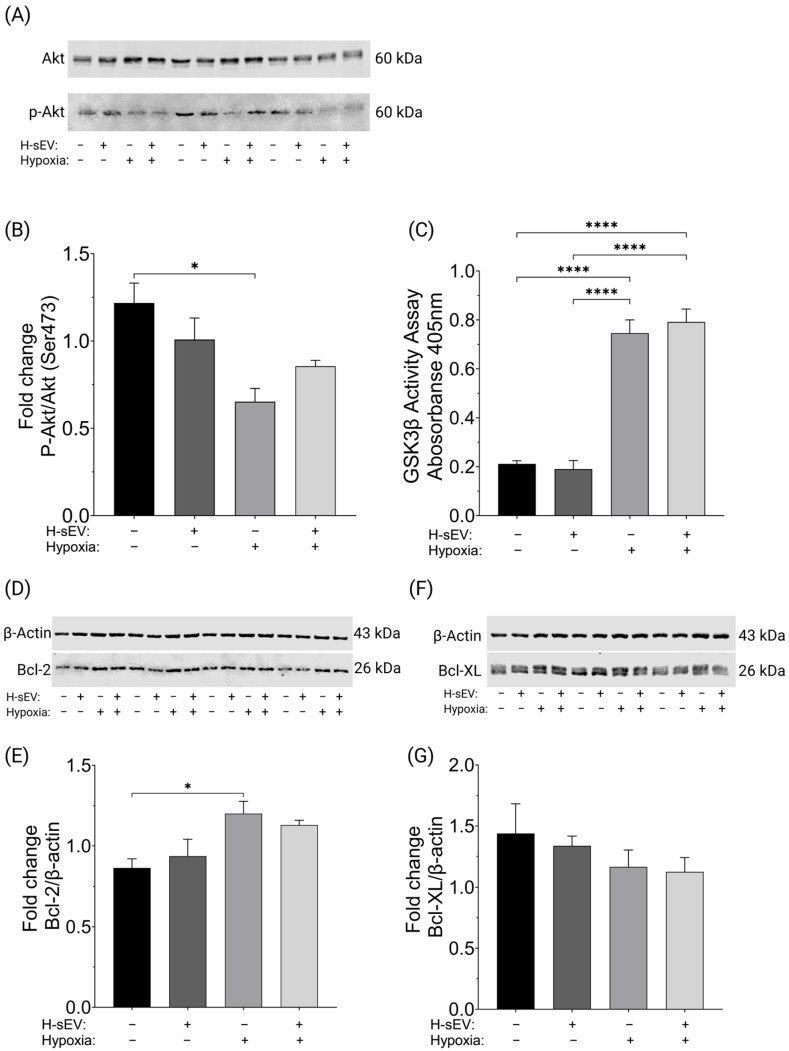
Regulation of key proteins involved in reperfusion injury salvage kinase (RISK) pathway. Western blotting and activity assay of the important RISK pathway mediators Protein Kinase B (Akt) and Glycogen synthase kinase-3β (GSK3β), as well as expression rates of the anti-apoptotic proteins B cell lymphoma-2 (Bcl-2) and B cell lymphoma extra-large (Bcl-XL). (**A**) Representative Western blots of Akt and *p*-Akt. (**B**) Western blotting shows that H-sEV preconditioning did not significantly affect Akt activation (*p*-Akt/Akt) in hypoxic CMs. (**C**) GSK3β activity assay shows that GSK3β activity increases after 18 h of hypoxia (+285%). However, H-sEVs did not significantly affect GSK3β activity after 18 h of hypoxia. (**D**) Representative Western blots of Bcl-2 normalized with β-Actin. (**E**) Western blotting shows that 18 h of hypoxia significantly increase Bcl-2 expression in control AC16 CMs. But 18 h of hypoxia did not significantly affect Bcl-2 expression in H-sEV-preconditioned cells. (**F**) Representative Western blots of Bcl-XL normalized with β-Actin. (**G**) Western blotting shows that Bcl-XL expression was not significantly changed after 18 h of hypoxic stimulation. Furthermore, H-sEV preconditioning had no affect on Bcl-XL expression compared with control cells. Protein expressions were normalized to loading control β-Actin in the densiometric analyses. Samples were evenly spread throughout the gel to account for data skewing based on gel placement. Data was analyzed using one-way ANOVA multiple comparison analysis. Data from the Western blotting and activity assay is expressed as mean of raw values ± SEM (*n* = 4). * *p* ≤ 0.05; **** *p* ≤ 0.0001.

Data from the caspase-3 assays revealed that hypoxia and H-sEV preconditioning negatively affect AC16 CMs. To further elucidate the underlying signaling processes causing these negative effects, we assessed protein regulation and activation of key factors involved in the RISK pathway, i.e., Protein Kinase B (Akt) (Ser473) [35] and GSK3β (Ser9). Western blotting analysis of Akt and phosphorylated Akt at Ser473 (*p*-Akt) (Figure 4A,B) showed that 18 h of hypoxia decreased levels of *p*-Akt in control AC16 CMs. However, 18 h of hypoxia did not significantly change *p*-Akt expression in H-sEV-preconditioned cells. Furthermore, H-sEV preconditioning did not significantly impact *p*-Akt expression compared to control AC16 CMs after 18 h of hypoxia. In addition, we performed a GSK3β activity assay to assess the impact of 18 h hypoxia and H-sEV preconditioning on the activation/inhibition of GSK3β (Figure 4C). Our results showed that 18 h of hypoxia drastically increased GSK3β activity (+285%, *p* ≤ 0.0001). Moreover, 24 h of preconditioning with H-sEVs did not alter GSK3β activity after 18 h of hypoxia, compared with control cells. To further investigate the intrinsic apoptotic pathway, caused by H-sEV preconditioning, we sought to determine the expression levels of the anti-apoptotic Bcl-2 family, i.e., B cell lymphoma-2 (Bcl-2) and B cell lymphoma extra-large (Bcl-XL). Bcl-2 and Bcl-XL negatively regulate Cytochrome C release from mitochondria to cytosol. We therefore measured their expression levels via Western blotting. The results of Western blotting analysis indicate a notable increase in Bcl-2 expression (Figure 4D,E) following 18 h of hypoxic stimulation in control AC16 CMs. Hypoxic stimulation did not significantly alter Bcl-2 expression in H-sEV-preconditioned cells compared to control cells. Moreover, Western blotting revealed that Bcl-XL expression (Figure 4F,G) remained unchanged following 18 h of hypoxia. In addition, H-sEV preconditioning did not impact Bcl-XL expression when compared to control cells.

### 2.5. Pro-Apoptotic Mediator of Intrinsic and Extrinsic Apoptosis

**Figure 5:**
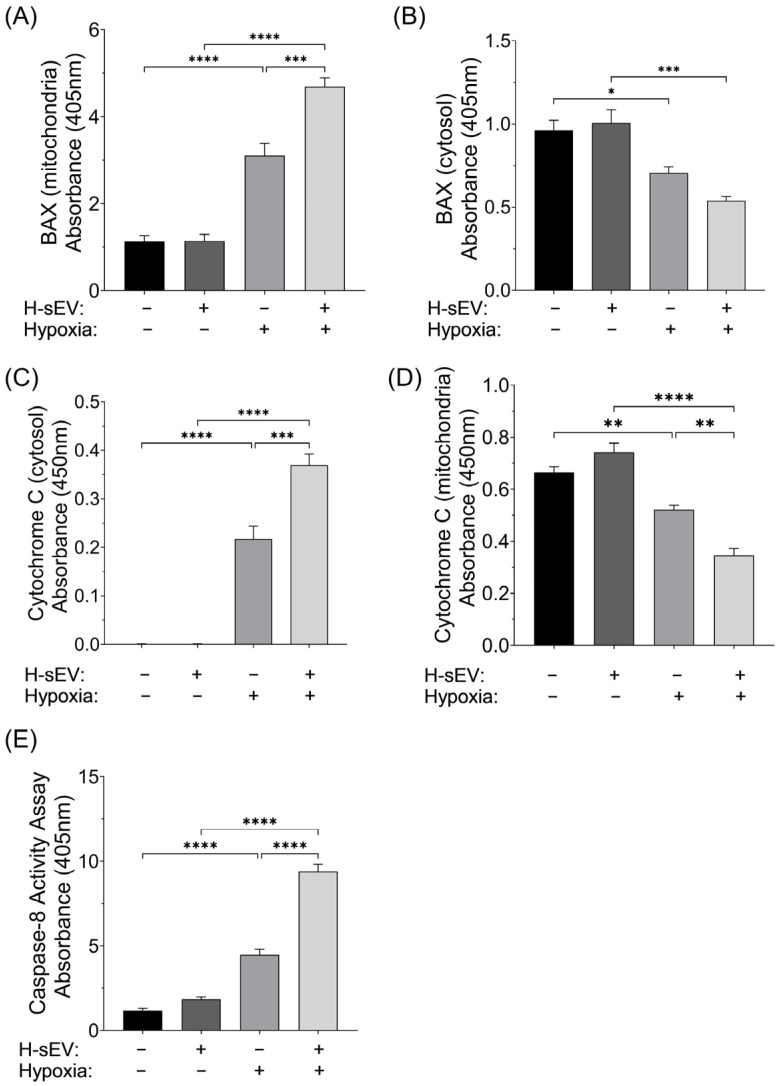
Translocation of BAX to mitochondria, release of cytochrome C, and activation of caspase-8. (**A**) Quantitative sandwich ELISA immunoassay showed a significant increase in Bcl-2-associated X (BAX) within the mitochondrial fraction of hypoxic cardiomyocytes (CMs). Moreover, hypoxic CMs preconditioned with H-sEVs displayed significantly higher (+51.6%) mitochondrial levels of BAX compared with hypoxic control cells. (**B**) Inversely, the cytosolic levels of BAX were significantly lowered after 18 h of hypoxia. There were no significant differences in cytosolic BAX levels between the two hypoxic groups. (**C**) Quantitative sandwich ELISA immunoassay showed a significant increase in Cytochrome C within the cytosolic fraction of hypoxic CMs. Interestingly, 24 h preconditioning with H-sEVs significantly increased Cytochrome C translocation (+70%) compared with control cells. (**D**) Inversely, the mitochondrial levels of Cytochrome C were significantly reduced in the hypoxic CMs. In addition, hypoxic CMs preconditioned with H-sEVs displayed significantly lower levels of Cytochrome C (−32.7%) within the mitochondrial fractions compared with hypoxic control cells. (**E**) Caspase-8 activity assay showed that 18 h of hypoxia increase caspase-8 activity in AC16 CMs. Furthermore, preconditioning with H-sEVs significantly increased caspase-8 activity (+110%) compared with control cells. To validate the integrity of the mitochondrial and cytosolic fractions, we performed several quantitative sandwich ELISA immunoassays. The mitochondrial fractions were validated by the absence of β-Actin and the presence of Cytochrome C Oxidase Subunit 4 (COX4), while the cytosolic fractions were validated by the presence of β-Actin and the absence of COX4 (Figure S1). Data was analyzed using one-way ANOVA multiple comparison analysis. Data is expressed as mean of raw values ± SEM (*n* = 4). * *p* ≤ 0.05; ** *p* ≤ 0.01; *** *p* ≤ 0.001; **** *p* ≤ 0.0001.

**Figure 6:**
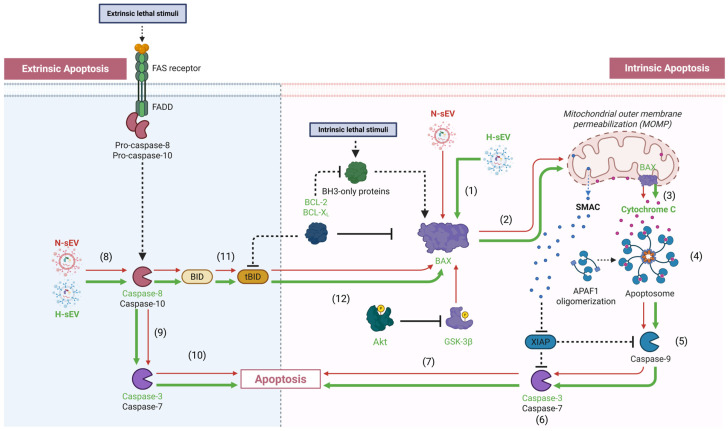
An illustrative model of observed effects from normoxic small extracellular vesicles (N-sEVs) and hypoxic small extracellular vesicles (H-sEVs) that mediated preconditional effects on the intrinsic and extrinsic apoptotic pathways of hypoxic stress. (1) H-sEV preconditioning (2) increased Bcl-2-associated X protein (BAX) translocation into the mitochondria, (3) resulting in increased translocation of Cytochrome C into the cytosol. (4) Increased levels of cytosolic Cytochrome C initiate the formation of the apoptosome complex (not determined in this study). (5) Apoptosome formation translates into increased caspase-9 activation (not determined in this study), (6) which subsequently leads to increased caspase-3 activation. (7) Once activated, caspase-3 induces apoptosis. (8) H-sEVpreconditioning did also increase caspase-8 activity in hypoxic CMs. (9) Caspase-8 can via the extrinsic apoptotic pathway directly induce caspase-3 activation, (10) resulting in apoptosis. In addition, caspase-8 can impact the intrinsic pathway of apoptosis by (11) cleaving BID into its truncated and active form tBID (not determined in this study), (12) which can lead to BAX activation. Proteins not investigated in this study are noted in black, while interactions not investigated are shown with dashed arrows. Observed effects of N-sEV and H-sEV preconditioning are illustrated with red and green arrows, respectively. The width of the arrows represents degree of observed effect.

In opposition to Bcl-2 and Bcl-XL, the pro-apoptotic protein BAX can translocate from the cytosol to mitochondria, where it increases mitochondrial permeability and subsequent Cytochrome C release [36]. We therefore isolated and separated the mitochondrial and cytosolic fractions of BAX to further elucidate the molecular changes facilitated by H-sEV preconditioning. BAX translocation was evaluated by measuring the mitochondrial and cytosolic levels of BAX via quantitative sandwich ELISA immunoassays. The results displayed a significant increase in BAX within the mitochondrial fractions of all hypoxic CMs (*p* ≤ 0.0001) (Figure 5A). Interestingly, the mitochondrial levels of BAX in the hypoxic CMs preconditioned with H-sEV were significantly higher compared with the hypoxic control cells (+51.6%, *p* ≤ 0.001). Moreover, the results showed a significant reduction in BAX within the cytosolic fractions of hypoxic CMs (Figure 5B). There were no significant differences in cytosolic BAX levels between the two hypoxia groups. Moreover, Cytochrome C release from mitochondria into the cytosol leads to the formation of the apoptosome complex and subsequent downstream activation of caspase-3 [37]. By measuring the cytosolic and mitochondrial levels of Cytochrome C using sandwich ELISA immunoassays, we found a significant increase in Cytochrome C within the cytosolic fractions of all cells stimulated with 18 h of hypoxia (*p* ≤ 0.0001) (Figure 5C). Furthermore, CMs preconditioned with H-sEVs displayed significantly increased Cytochrome C translocation compared with control cells post hypoxia (+70%, *p* ≤ 0.001). We detected no abundance of Cytochrome C within the cytosolic fractions of normoxic preconditioned or normoxic control cells. Moreover, we detected a significant reduction in Cytochrome C within the mitochondrial fractions of hypoxic CMs (Figure 5D). In addition, our results showed that CMs preconditioned with H-sEVs displayed significantly decreased levels of mitochondrial Cytochrome C compared with control CMs post hypoxia (−32.7%, *p* ≤ 0.01). Next, we sought to determine the impact of hypoxia and H-sEV preconditioning on the extrinsic apoptotic pathway of caspase-3 activation. Thus, we investigated the impact of H-sEV preconditioning on caspase-8 activity (Figure 5E). Our results showed that 18 h of hypoxia greatly increased caspase-8 activity for both H-sEV-preconditioned and control AC16 CMs. However, AC16 CMs preconditioned with H-sEVs displayed significantly increased caspase-8 activity compared to control CMs after 18 h of hypoxia (+110%, *p* ≤ 0.0001). An illustrative model summarizing the observed preconditioning effects of normoxia-derived sEVs (N-sEVs) and H-sEVs on the regulation of intrinsic and extrinsic apoptotic pathways in cells exposed to hypoxic stress is presented in Figure 6.

## 3. Discussion

The present study sought to investigate the effects of sEV-mediated cell-cell signaling between CMs during hypoxia. To accomplish this, we applied a preconditional approach, isolating sEVs from the culture media of hypoxic AC16 CMs, before administrating them to a parallel set of AC16 CMs pre-18 h of hypoxia. Hypoxic stimulation was initially validated by the expression of HIF-1α. Following this, we investigated key signaling pathways involved in regulation of apoptosis.

In our study, we observed a significant increase in caspase-3 activity following hypoxic conditions. This increase was further exacerbated in CMs that were preconditioned with H-sEVs. The augmented caspase-3 activity suggests that H-sEVs trigger apoptotic signaling processes, thereby reducing the resistance of CMs to hypoxia. To shed light on the molecular mechanisms that underpin the adverse effects of H-sEV preconditioning and hypoxic stimulation, we carried out a series of experiments. These were designed to identify the regulation and activation of key factors within the RISK pathway. This approach allowed us to gain a deeper understanding of the processes involved. The protein kinase Akt has been reported to be a prominent modulator of the apoptotic pathway in response to hypoxia [38]. Once phosphorylated at Ser473, Akt activates downstream pro-survival cascades. Our results demonstrated that 18 h of hypoxic stress resulted in decreased levels of *p*-Akt in control AC16 CMs. No further effect was observed by H-sEV treatment.

GSK3β is also considered an important signaling mediator involved in the cellular response to hypoxia [20]. GSK3β is inactivated upon phosphorylation at Ser9 and can facilitate cell survival or apoptosis dependent on its phosphorylated state [39,40]. In its active state, GSK3β promotes mitochondrial outer membrane permeabilization and initiation of intrinsic apoptotic pathways [41]. In contrast, phosphorylation of GSK3β induces a cardioprotective response [17]. Here we found that GSK3β activity was greatly increased after hypoxia. However, we did not observe any differences in GSK3β activity between H-sEV-preconditioned and control cells. These data indicate that the observed caspase-3 increase following H-sEV stimulation was not related to altered GSK3β activity.

To explain the increased caspase-3 activity observed with H-sEVs, we sought to quantify the expression levels of the pro-apoptotic protein BAX. Once activated, BAX translocates from the cytosol into the mitochondria where it, together with BAK, forms the MAC pore complex [36], resulting in Cytochrome C release to the cytosol. Our data clearly demonstrated that BAX is recruited from the cytosolic pool to the mitochondria during hypoxia, a process that was significantly amplified by treatment with H-sEVs. It should be noted that our data shows a disproportional rise of measured BAX levels within the mitochondrial fraction compared to the depleted levels of measured BAX within the cytosolic fractions. BAX can bind to proteins and complexes forming higher molecular weight domains, which might not be fully solubilized by standard cytosolic fractioning, resulting in an unmeasured cytosolic pool [42,43].

To further elucidate the intrinsic pathway of caspase-3 activation and apoptosis, we next investigated Cytochrome C release from the mitochondria into the cytosol. In accordance with the increased BAX recruitment to the mitochondria, hypoxic CMs exhibited significantly higher cytosolic Cytochrome C levels. Furthermore, CMs treated with H-sEVs displayed significantly worse outcomes with even higher levels of cytosolic Cytochrome C compared to control. Combined, the data from BAX, Cytochrome C release from the mitochondria, and activation of caspase-3 clearly indicate that preconditioning with H-sEVs increases apoptosis. Increased Cytochrome C release can also be explained by reduced activity of anti-apoptotic proteins during hypoxia-induced stress. However, we did not see any impact of H-sEV treatment on either Bcl-2 or Bcl-XL during hypoxia.

After revealing H-sEVs mediated activation of the intrinsic pathway of caspase-3 activation, without impacting GSK3β activity or Bcl-2 and Bcl-XL, we sought to elucidate whether H-sEV preconditioning could affect the extrinsic pathway of apoptosis as well. Caspase-3 can be activated via death ligand–death receptor-mediated interactions, e.g., Tumor necrosis factor alpha (TNF-α)/Tumor necrosis factor receptor 1 (TNFR1) [44] or Fatty acid synthetase ligand/Fatty acid synthetase receptor [45]. Once ligand–receptor binding occur, adaptor proteins are recruited and bind to the complex, e.g., Fas-associated death domain (FADD), TNF receptor-associated death domain, and Receptor-interacting protein [46]. The adaptor proteins then form a death-inducing signaling complex with procaspase-8, which subsequently is cleaved into its active form caspase-8 [47]. Caspase-8 is a key initiator of the extrinsic apoptotic pathway and functions upstream of caspase-3 activation. It is primarily activated through death receptor-mediated signaling cascades in response to extracellular signals, such as binding of death ligands to their receptors [48]. Caspase-8 can also interact with the intrinsic pathway by cleaving BID into its active form tBID, which promotes mitochondrial Cytochrome C release and subsequent caspase-3 activation [37,49]. In our study, 18 h of hypoxia significantly increased caspase-8 activity, and this effect was further pronounced in cells preconditioned with H-sEVs compared to hypoxic controls. These findings align with the observed increase in caspase-3 activity and indicate that H-sEV preconditioning is associated with activation of both intrinsic and extrinsic apoptotic pathways under hypoxic conditions.

Although a broad amount of research supports the notion that H-sEVs promote cardiac repair and cell survival [50,51], sEVs derived from hypoxic CMs have also been reported to increase apoptosis and negatively impact cardiac cells. Lin, B. et al. [52] reported that sEVs secreted from hypoxic AC16 CMs carried the lncRNA HCG15. Their study concluded that the presence of hypoxic sEVs causes apoptosis in CMs, increased levels of inflammatory factors, and suppressed cellular proliferation through the NF-κB/p65 and p38 pathways. Similarly, Wang, L. et al. [53] have demonstrated that sEVs secreted from adult rat CMs carry lncRNA AK139128, which promote apoptosis and inhibit cellular proliferation in recipient cardiac fibroblasts, both in vitro and in vivo. Furthermore, in a study published by Yang, Y. et al. [54], sEVs secreted from hypoxic CMs in vitro were proposed to compromise cardiac function by the transfer of miR-30a.

In a previous study, we reported potentially protective effects of exosomes, shown by increased beat period and improved excitation–contraction coupling when preconditioning hypoxic hIPSC-CMs [55]. However, that study did not assess apoptotic signaling, which could also occur alongside electrophysiological changes. Differences likely reflect distinct endpoints—electrophysiology versus apoptosis—and cell type characteristics. These observations suggest that sEV effects are context-dependent and warrant further investigation.

This study primarily focused on examining the functional effects of H-sEVs on apoptotic signaling in AC16 CMs. We recognize that a comprehensive analysis of H-sEV cargo relative to N-sEVs would substantially strengthen mechanistic interpretation of our findings. Future studies are required to identify specific enriched proteins, lipids, or RNA within the H-sEVs. Such work should include proteomics and small RNA profiling, followed by targeted validations.

AC16 CMs were chosen as the cellular model for this study as they are a non-excitable, immortalized human cell line capable of proliferation and differentiation. In addition, they are known to express key CM markers [56], making them a useful in vitro model for investigating pathological mechanisms and stress responses relevant to IHD [57,58,59]. AC16 CMs have also previously been used to study sEV biology [52]. Moreover, while primary CMs from animal models provide valuable insights into cardiac disease, their genetic background does not accurately reflect that of human CMs. Additionally, the acquisition and maintenance of primary human CMs remains technically challenging.

In conclusion, we investigated sEV-mediated signaling in hypoxic CMs. Using an H-sEV preconditioning approach, we focused on hypoxia stress response and apoptosis pathways. We showed that hypoxic AC16 CMs display increased HIF-1α expression, significant increase in both caspase-3 and caspase-8 activity, as well as increased BAX recruitment to the mitochondria with consequences of mitochondrial Cytochrome C release. As H-sEVs had no significant impact on the HIF-1α expression in hypoxic AC16 CMs, these observed effects appear to be independent of HIF-1α. While sEVs have traditionally been associated with cardiac repair and cell survival, preconditioning AC16 CMs with H-sEVs resulted in increased BAX activation and Cytochrome C release, ultimately leading to increased caspase-3 activity and apoptosis. Interestingly, this effect was not associated with altered Akt or GSK3β activity, indicating an independent pathway for H-sEV-mediated activation of caspase-3. Additionally, H-sEV preconditioning further increased caspase-8 activity, implicating additional involvement of H-sEVs on the extrinsic pathway of apoptosis. These findings underscore the complexity of sEV signaling and provide insights into hypoxia-related cardiac diseases. Understanding these mechanisms could aid in developing potential therapies.

## 4. Materials and Methods

### 4.1. AC16 CM Culture and Maintenance

AC16 CM human cells (obtained from EMD Millipore/Merck Life Sciences, #SCC109, Darmstadt, Germany) were cultured and seeded in 100 mm dishes treated for cell culture (Thermo Scientific™, #130182, Waltham, MA, USA). The maintenance medium used was a 1:1 (*v*/*v*) mix of Dulbecco’s modified Eagle’s medium (DMEM) and Ham’s F12, supplemented with 2 mM Glutamine, 12.5% Fetal Bovine Serum, and 1% antibiotic/antimycotic mix, as per the instructions given by the supplier. The AC16 CM cells were kept in a humidified atmosphere with 21% O_2_ and 5% CO_2_ at 37 °C, as outlined in previous work [57]. The cells were passaged every 4–5 days and utilized within the P1–P10 range, adhering to the provider’s recommendations.

### 4.2. Generation of sEVs from Normoxic and Hypoxic AC16 CMs

sEVs were generated from AC16 CMs (70–85% confluency) exposed to either normoxia: 6 h incubation with maintenance medium (with sEV-depleted fetal bovine serum) at 21% O_2_, 5% CO_2_ in a humidified atmosphere at 37 °C, or hypoxia: 6 h incubation with hypoxia medium (Table S1), at 1% O_2_, 5% CO_2_, 94% N_2_ in a humidified atmosphere at 37 °C. Hypoxia challenge was induced and maintained for the designated duration using the New Brunswick™ Galaxy^®^ 48 R CO2 incubator (Eppendorf Norge AS, Oslo, Norway). The specific parameters of the hypoxic challenge, i.e., the use of a defined hypoxia medium and controlled low-oxygen incubation, were selected to closely mimic ischemic conditions, as previously described [60]. Following incubation, sEVs were isolated from the condition medium of the normoxic and hypoxic AC16 CMs. sEVs isolated from normoxic and hypoxic condition medium are hence referred to as N-sEVs and H-sEVs, respectively.

### 4.3. Isolation and Resuspension of AC16 CM-Derived sEVs

sEV isolation was carried out using the Total Exosome Isolation Reagent (from cell culture medium) (Invitrogen^TM^, #4478359, Waltham, MA, USA), in accordance with the protocol provided by the manufacturer. First, conditioned medium (either from normoxic or hypoxic conditions) was collected from the cell culture dish and centrifugation at 2000× *g* for 30 min. After collecting the supernatant in new tubes, 0.5 volumes of the isolation reagent were added, mixed, and left for overnight incubation at 2 °C to 8 °C. Next day, samples underwent centrifugation at 10,000× *g* for 1 h at 2 °C to 8 °C. After centrifugation, the supernatant was removed and discarded, leaving behind the sEV pellet, which was then resuspended in maintenance medium. sEVs were resuspended in equal volumes as first aspirated from the CMs, ensuring a 1:1 transfer ratio of sEVs. sEVs were used immediately following resuspension.

### 4.4. sEV Quantification and Characterization

The size distribution and concentration of AC16 CM-secreted sEVs were assessed by Nanoparticle Tracking Analysis (NTA). A NanoSight NS300 (Malvern Instruments Ltd., Worcestershire, UK) instrument was used to perform the NTA. A dilution of 1:100 was used to ensure accurate measurements of the samples. Each sample was measured 5 times during 60 s intervals for each sample (*n* = 4). These settings were used, in accordance with the guidelines set by the manufacturer (NanoSight NS300 User Manual, MAN0541-01-EN-00, 2017, Malvern Instruments Ltd., Worcestershire, UK): Hardware: Camera; Type: sCMOS; Laser Type: Blue405. Camera level was set to 12, and the manual focus was adjusted to clearly visualize the particles. The analysis conditions included a temperature set to 21 °C and a syringe pump speed of 70. Data was processed using NanoSight Software NTA 3.2 Dev Build 3.2.16, with a detection threshold of 8. As advised by the guidelines, we ensured the number of particles counted in each measurement exceeded the recommended minimum.

To confirm the presence of known sEV protein markers in pooled isolated particle samples (*n* = 6), we utilized the Exo-Check™ Exosome Antibody Array (System Bioscience, # EXORAY200B-4, Palo Alto, CA, USA) following the manufacturer’s protocol. This kit includes eight antibodies targeting known sEV markers (CD63, CD81, ALIX, FLOT1, ICAM1, EpCam, ANXA5, and TSG101), along with two positive controls, one blank, and an antibody for the cis-Golgi matrix protein (GM130). The protein concentration of the pooled isolate was first measured using the Pierce BCA Protein Assay (Thermo Scientific, #23227, Waltham, MA, USA). A total of 50 µg of protein was applied to the antibody array. The pooled sEV isolate was lysed and labeled with 1 µL of labeling agent, followed by a 30-min incubation. Excess labeling agent was removed, and the labeled pooled sEV isolate was mixed with blocking buffer before being added to the antibody-coated membrane. The membrane was incubated with the labeled sample overnight at 4 °C. The following day, it was washed with the provided washing buffer and incubated for 30 min with detection reagents. Afterward, the membrane underwent three additional washes before imaging. SuperSignal West Pico PLUS Chemiluminescent Substrate (Thermo Scientific, #34580, Waltham, MA, USA) served as the HRP developer solution. Imaging was performed using the LI-COR Odyssey XF imaging system (LI-COR Bioscience, Lincoln, NE, USA) with an exposure time of 5 min.

### 4.5. sEV Preconditioning

For preconditioning, H-sEVs resuspended in CM Culture Media were given to AC16 CMs seeded in 100 mm Cell Culture Treated Dishes (70–85% confluency). The cells received H-sEVs (7 mL, 7.6 × 10^7^ sEV/mL) before 24 h incubation at 21% O_2_, 5% CO_2_ and at 37 °C. To account for trace amounts of the sEV isolation reagent, N-sEVs were also isolated and given to cells as a preconditional treatment. Cells preconditioned with N-sEVs were used as control groups and will henceforth be referred to as control cells.

### 4.6. Post-Preconditional Normoxic or Hypoxic Stimulation

Immediately after sEV preconditioning, the AC16 CMs were exposed to two different conditions. For the normoxic condition, cells were incubated with maintenance medium at 21% O_2_, 5% CO_2_ and 37 °C in a humidified atmosphere for 18 h. For the hypoxic condition, cells were incubated with hypoxia medium (Table S1), at 1% O_2_, 5% CO_2_, 94% N_2_ at 37 °C in a humidified atmosphere for 18 h.

### 4.7. Quantitative Measurement of HIF-1α

The levels of HIF-1α in the experimental lysates were determined by sandwich ELISA immunoassay as previously described [61]. Briefly, HIF-1α capture antibody (30 ng, details in Table 1) was immobilized in each well of the 96-well microplate. The respective experimental lysates (input), equivalent to 10 µg of protein content, were incubated with the immobilized HIF-1α capture antibody overnight at 4 °C. The conditioned lysates were then discarded, and the 96-well microplate wells were washed three times for 15 min each with TBS-T and incubated with the HIF-1α detection antibody (Table 1) overnight at 4 °C. The 96-well microplate wells were again washed with TBS-T, followed by immunodetection with the HRP-conjugated secondary antibody, using the HRP substrate TMB (3,3′,5,5′-tetramethylbenzidine) (Thermo Fisher Scientific, Oslo, Norway, Catalogue #N301) as a chromophore for the colorimetric read-out (λ_450_). The antibody signal specificity was established by performing antibody-blocking assays in the entire spectrum of experimental lysates. The antibody-blocking peptide corresponding to the specific epitope for the HIF-1α detection antibody used is depicted in Table 1. The respective absorbances from the antibody-blocking assays were used for experimental blank correction. Data is expressed as blank-corrected absorbances from three technical replicates for each of the four biological replicates belonging to each experimental group.

### 4.8. PrestoBlue Cell Viability Assay

A PrestoBlue cell viability assay was used to measure the effects of 18 h of hypoxia and H-sEV preconditioning on the cell viability of AC16 CMs. Firstly, AC16 CMs were seeded in 96-well culture plates (5000 cells/well) and maintained with maintenance medium to 70–85% confluency. Once confluent, cells were subjected to N-sEV or H-sEV preconditioning, directly followed by normoxic or hypoxic stimulation. An amount of PrestoBlue™ Cell Viability Reagent (Invitrogen™, # A13261, Waltham, MA, USA) was used following the manufacturer’s instructions. Cells were incubated with the PrestoBlue reagent for 2 h at 37 °C to increase assay sensitivity. PrestoBlue cell viability data are expressed as experimental blank-corrected values (absorbance 570 nm) from three technical replicates representing 4 distinct experimental groups each with 6 biological replicates (*n* = 6).

### 4.9. Caspase-3 Activity Assay

Caspase-3 activity was measured spectrophotometrically using the caspase-3 substrate Ac-DEVD-p-NA (N-Acetyl-Ac-Asp-Glu-Val-Asp-p-nitroanilide) (Sigma Aldrich, # 235400-5MG, Oslo, Norway), as previously described [57]. In short, the activity of caspase-3 was gauged as an indicator of the quantity of the chromophore released due to proteolysis by caspase-3. AC16 CM cells, initially maintained in 100 mm culture plates under specific conditions, were trypsinized and centrifuged at 1000× *g* for 5 min. The cell pellets were lysed with a buffer (50 mM HEPES, 5 mM CHAPS, pH 7.4) and incubated on ice (10 min). After centrifugation at 12,000× *g* for 15 min to pellet the cell debris, the supernatant with non-denatured cell lysate was transferred to new tubes serving as input for the caspase-3 activity quantification. The samples were incubated with the caspase-3 substrate Ac-DEVD-p-NA (200 µM). To confirm assay specificity, a caspase-3 activity assay was also performed with the caspase-3 inhibitor Ac-DEVD-CHO. The absorbance of the chromophore produced by caspase-3 was measured at 405 nm. Absorbances from the inhibited samples were used for blank correction. Caspase-3 activity data are presented as blank-corrected absorbance values (405 nm) from three technical replicates across four experimental groups, each with four biological replicates.

### 4.10. Western Blotting in AC16 CMs

In addition to sEV characterization, Western blotting analysis was used to determine the expression levels of important modulators of the cellular responses to hypoxia and cell death. Similar as for sEVs, protein isolation for AC16 CM whole lysate was performed using standard protocols [2]. Once proteins (10–50 µg) were resolved on SDS-PAGE gels, they were transferred onto a PVDF membrane via semidry blot (Trans-Blot Turbo Transfer System, #1704150, Bio-Rad, Hercules, CA, USA). Membranes were blocked with Intercept^®^ Blocking Buffer (LI-COR Bioscience, #927-60001, Lincoln, NE, USA) for 1 h before overnight incubation with the respective primary antibodies at 4 °C following standardized protocols [62]. The origin, source, dilutions of the respective antibodies used in this study are compiled in Table 1. β-Actin or post-transcriptional modifications were used as a gel loading control for whole-cell lysates. The Western blots were developed with IRDye^®^ Secondary Antibodies (LI-COR Bioscience, Lincoln, NE, USA), and imaged using a LICOR Odyssey XF imaging system (LI-COR Bioscience, Lincoln, NE, USA). Quantification of results was performed by densitometry using Empira Studios 2.2 (LI-COR Bioscience, Lincoln, NE, USA), and the results were analyzed as total integrated densitometric values.

### 4.11. GSK3β Kinase Activity Assay

GSK3β activity was measured in non-denatured AC16 CM lysates using an indirect ELISA immunoassay protocol, as previously described in detail [40]. Briefly, the phosphorylation of a synthetic peptide’s serine residue (RRRPASVPPSPSLSRHS(pS)HQRR) was used as a stand-in for the GSK3β target muscle glycogen synthase 1. This phosphorylation serves as an indirect measure of GSK3β kinase activity. Streptavidin (110 µL, 20 pmoles, pH 6.5) was used to coat 96-well cell culture plates. The GSK3β substrate (GSM, Sigma Aldrich/Merck Life Science, #12-533, Burlington, VT, USA) was biotinylated using the “EZ-Link™ NHS-LC-Biotin” kit (Thermo Fisher Scientific, #21336, Waltham, MA, USA). AC16 CM cell lysate (50 µg) was then incubated on these plates overnight at 4 °C. The next day, samples were incubated with a specific primary antibody (100 ng/well, 50 µL of 2 µg/mL) against the phosphorylated serine residue for 24 h. Phosphorylated serine residues were detected using an AP-conjugated secondary antibody and the AP-substrate p-nitrophenyl phosphate disodium salt for colorimetric measurements (λ_405_). To confirm assay specificity, the GSK3β kinase activity assays were also performed with the GSK3β kinase inhibitor SB-216763 (Sigma Aldrich/Merck Millipore/Merck Life Science, #S3442, Darmstadt, Germany). The optical density values from the inhibitor-treated lysates were used for blank correction. GSK3β activity data are presented as blank-corrected absorbance values (405 nm) from three technical replicates across four experimental groups, each with four biological replicates.

### 4.12. Cellular Fractionation to Segregate the Cytosolic and Mitochondrial Compartments

To segregate the cellular cytosolic and mitochondrial fraction, the “Mitochondria/Cytosol Fractionation Kit” (Abcam, # ab65320, Cambridge, UK) was used. The isolation procedures were performed according to previous operating procedures [40].

### 4.13. Validity and Integrity of the Fractionated Mitochondrial and Cytosolic Compartments Subjected to Cytochrome C Release Assay and the Quantitative Sandwich ELISA Immunoassays of BAX

The integrity of the mitochondrial fractions was validated by the absence of β-Actin and the presence of COX4, while the cytosolic fractions were validated by the presence of β-Actin and the absence of COX4 determined via quantitative sandwich ELISA immunoassay according to previously published procedures [40]. In short, capture antibodies were immobilized in a 96-well microplate, and cytosolic and mitochondrial fractions were added and incubated overnight at 4 °C. After washing with TBS-T, detection antibodies were added and incubated overnight at 4 °C. Following another wash, immunodetection was performed using an HRP-conjugated secondary antibody and the HRP-substrate Amplex Red (10-acetyl-3,7-dihydroxyphenoxazine) (Thermo Fisher Scientific, # A22188, Oslo, Norway). The signal was measured at λ_570_. Parallel assays with blocking peptides ensured antibody and signal specificity (Table 1). Absorbances from these assays were used for blank correction. Data are presented as blank-corrected absorbance values (570 nm) from three technical replicates across four experimental groups, each with four biological replicates.

### 4.14. Quantitative Measurement of the Pro-Apoptotic Protein BAX

To measure BAX levels in cytosolic and mitochondrial fractions, we used a quantitative sandwich ELISA as previously described [61]. Briefly, BAX capture antibody (20 ng, details in Table 1) was immobilized in a 96-well microplate. Cytosolic (40 µg protein) and mitochondrial (20 µg protein) fractions were added and incubated at 4 °C overnight. After incubation, the wells were washed with Tris-buffered saline with 0.1% *v*/*v* Tween-20 (TBS-T) three times for 15 min each. A BAX detection antibody was then added and incubated overnight at 4 °C. Following another wash with TBS-T, immunodetection was performed using an HRP-conjugated secondary antibody using the HRP-substrate o-phenylenediamine dihydrochloride (Thermo Fisher Scientific, Oslo, Norway, Catalogue # 34005). The signal was measured at 450 nm. To ensure specificity, parallel assays with blocking peptides were conducted, and absorbances from these assays were used for blank correction. BAX expression data were reported as blank-corrected absorbance values (450 nm) from three technical replicates across four experimental groups, each with four biological replicates.

### 4.15. Cytochrome C Release Assay

The rate of Cytochrome C translocation from the mitochondria into the cytosol was determined by analyzing the relative abundance of Cytochrome C in the cytosolic fraction versus the mitochondrial fraction. The abundance of Cytochrome C in the respective fractions was measured as previously described [57], using a quantitative sandwich ELISA immunoassay approach (antibody details in Table 1). Cytochrome C expression data are expressed as experimental blank-corrected values (absorbance 450 nm) from three technical replicates representing 4 distinct experimental groups each with 4 biological replicates (*n* = 4).

### 4.16. Caspase-8 Activity Assay

In a manner akin to the caspase-3 activity assay, we quantified caspase-8 activity using spectrophotometry. The specific substrate for caspase-8, Ac-IETD-pNA (Acetyl-Ile-Glu-Thr-Asp-p-Nitroaniline), was procured from Sigma Aldrich (#A9968-5MG, Oslo, Norway). The measurement of caspase-8 activity served as an indirect measure of the quantity of the chromophore p-nitroaniline released due to the proteolytic action of caspase-8 on Ac-IETD-pNA, as detailed in our previous work [57]. The caspase-8 activity data, expressed as absorbance at 405 nm after correcting for experimental blank, were obtained from three technical replicates for each of the four biological replicates in each of the four distinct experimental groups (*n* = 4).

### 4.17. Statistical Analysis

The NTA data was analyzed using NanoSight Software NTA 3.2 Dev Build 3.2.16. For the statistical evaluation of the activity assay results, GraphPad Prism 9 (GraphPad Software, San Diego, CA, USA) was used. The significance of differences among the samples was determined by one-way ANOVA. Quantitative data for all assays are presented as mean ± SEM.

## Figures and Tables

**Table 1:** List of antibodies and antibody-blocking peptides.

Antibody	Application	Amount	Host	Manufacturer	Catalogue #	Resource Identifier ID (RRID)
Akt	Western Blot	1:1000 Dilution	Mouse	Cell Signaling Technology (Danvers, MA, USA)	2920	AB_1147620
Phospho-Akt (Ser473)	Western Blot	1:1000 Dilution	Rabbit	Cell Signaling Technology (Danvers, MA, USA)	9271	AB_329825
β-Actin	Western Blot	1:5000 Dilution	Mouse	Santa Cruz Biotechnology (Dallas, TX, USA)	sc-47778	AB_2714189
β-Actin	ELISA Capture	20 ng/well	Mouse	Santa Cruz Biotechnology (Dallas, TX, USA)	sc-47778	AB_2714189
β-Actin	ELISA Detection	20 ng/well	Rabbit	Cell Signaling Technology (Danvers, MA, USA)	4970	AB_2223172
β-Actin antibody-blocking peptide	ELISA Detection	N/A	N/A	Cell Signaling Technology (Danvers, MA, USA)	1025	N/A
BAX	ELISA Capture	20 ng/well	Mouse	Thermo Fisher Scientific (Oslo, Norway)	33-6600	AB_2533133
BAX	ELISA Detection	20 ng/well	Rabbit	Novus Biologicals (Centennial, CO, USA)	NBP1-88682	AB_11014342
BAX antibody-blocking peptide	ELISA Detection	N/A	N/A	Novus Biologicals (Centennial, CO, USA)	NBP1-88682PEP	N/A
Bcl-XL	Western Blot	1:1000 Dilution	Rabbit	Cell Signaling Technology (Danvers, MA, USA)	2764	N/A
Bcl-2	Western Blot	1:500 Dilution	Mouse	Thermo Fisher Scientific (Oslo, Norway)	BMS1028	AB_10597451
COX4	ELISA Capture	20 ng/well	Mouse	Thermo Fisher Scientific (Oslo, Norway)	MA5-15686	AB_10977841
COX4	ELISA Detection	20 ng/well	Rabbit	Cell Signaling Technology (Danvers, MA, USA)	4844	AB_2085427
COX4 antibody-blocking peptide	ELISA Detection	N/A	N/A	Cell Signaling Technology (Danvers, MA, USA)	1034	N/A
Cytochrome C	ELISA Capture	20 ng/well	Mouse	Thermo Fisher Scientific (Oslo, Norway)	BMS1037	AB_10598651
Cytochrome C	ELISA Detection	20 ng/well	Rabbit	Cell Signaling Technology (Danvers, MA, USA)	4280	AB_10695410
Goat Anti-Rabbit IgG (H + L)-HRP Conjugate	ELISA	N/A ^€^	Goat	Bio-Rad (Hercules, CA, USA)	1706515	AB_11125142
HIF-1α	ELISA capture	30 ng/well	Mouse	Thermo Fisher Scientific (Oslo, Norway)	MA1-16504	AB_568567
HIF-1α	ELISA detection	20 ng/well	Rabbit	Novus Biologicals (Centennial, CO, USA)	NBP1-47180	AB_10010137
HIF-1α antibody-blocking peptide	ELISA detection	N/A	N/A	Novus Biologicals (Centennial, CO, USA)	NBP1-47180PEP	N/A
IRDye^®^ 800CW Goat anti-Mouse IgG Secondary Antibody	Western Blot	1:20,000 Dilution	Goat	LI-COR (Lincoln, NE, USA)	926-32210	N/A
IRDye^®^ 800CW Goat anti-Rabbit IgG Secondary Antibody	Western Blot	1:20,000 Dilution	Goat	LI-COR (Lincoln, NE, USA)	926-32211	N/A
IRDye^®^ 680RD Goat anti-Mouse IgG Secondary Antibody	Western Blot	1:20,000 Dilution	Goat	LI-COR (Lincoln, NE, USA)	926-68070	N/A
IRDye^®^ 680RD Goat anti-Rabbit IgG Secondary Antibody	Western Blot	1:20,000 Dilution	Goat	LI-COR (Lincoln, NE, USA)	926-68071	N/A

N/A: Not Applicable. €: Amount of secondary antibody cannot be determined as the commercial vendor does not provide the antibody concentration.

## Data Availability

The original contributions presented in this study are included in the article/Supplementary Materials. Further inquiries can be directed to the corresponding author.

## Supplementary Materials

The following supporting information can be downloaded at: https://www.mdpi.com/article/10.3390/ijms262412123/s1.

## Abbreviations

Akt	protein kinase B
ATP	adenosine triphosphate
BAK	Bcl2-homologous antagonist/killer
BAX	Bcl-2-associated X protein
CM	cardiomyocyte
COX4	Cytochrome C Oxidase Subunit 4
EV	extracellular vesicle
GSK3β	glycogen synthase kinase-3β
H-sEV	hypoxia-derived small extracellular vesicle
HIF-1α	hypoxia-inducible transcription factor 1α
MAC	mitochondrial apoptosis-induced channel
miR	microRNA
N-sEV	normoxia-derived small extracellular vesicle
NTA	nanoparticle tracking analysis
RIPC	remote ischemic preconditioning
RISK	reperfusion injury salvage kinase
ROS	reactive oxygen species
