# Supplementary material for: Hypoxic Small Extracellular Vesicle Preconditioning of AC16 Cardiomyocytes Increase Caspase-3 and Caspase-8 Activity During Hypoxia
Source: Int J Mol Sci. 2025 Dec 17;26(24):12123. doi: 10.3390/ijms262412123 (PMC12733103; doi:10.3390/ijms262412123)
Supplement: Supplementary file 1 [file ijms-26-12123-s001.zip › ijms-4012381-supplementary.pdf]

Supplement

Table S1

**Component**	**Amount 20mL**	**Final concentration**		**50mL total**
DMEM, no Glucose, no Glutamine, no Phenol Red	18.578 mL	93%		46.45 mL
Creatine (hydrous)	13.12 mg	5 mM		32.8 mg
D-(+) Glucose Solution 2.5 M, 450 g/L	0.022 mL	2,75 mM		55uL
Glutamine 200 mM	0.2 mL	2 mM		500uL
HEPES 1M	0.2 mL	10 mM		500uL
L-carnitine 200 mM	0.2 mL	2 mM		500uL
Non-essential Amino Acids 100X	0.2 mL	1X		500uL
Sodium Pyruvate 100 mM	0.2 mL	1 mM		500uL
Taurine 500 mM	0.2 mL	5 mM		500uL
Linoleic-oleic Acid 100X (add after filtering the rest)	0.2 mL	1X		500uL

Supplementary Table S1: Notation legend

1. Thermo Fisher Scientific, Oslo, Norway, Catalogue # 11966025

2. Sigma Aldrich / Merck Millipore / Merck Life Science, Darmstadt, Germany, Catalogue # C3630-100G

3. Sigma Aldrich / Merck Millipore / Merck Life Science, Oslo, Norway, Catalogue # G8769

4. Thermo Fisher Scientific, Oslo, Norway, Catalogue # A2916801

5. Sigma Aldrich / Merck Millipore / Merck Life Science, Darmstadt, Germany, Catalogue # H4034-500G

6. Sigma Aldrich / Merck Millipore / Merck Life Science, Darmstadt, Germany, Catalogue # C0283-25G

7. Thermo Fisher Scientific, Oslo, Norway, Catalogue # 11140035

8. Thermo Fisher Scientific, Oslo, Norway, Catalogue # 11360070

9. Sigma Aldrich / Merck Millipore / Merck Life Science, Darmstadt, Germany, Catalogue # T8691-100G

10. Sigma Aldrich / Merck Millipore / Merck Life Science, Darmstadt, Germany, Catalogue # L9655-5ML

* N/A - Not Applicable

Figure S1

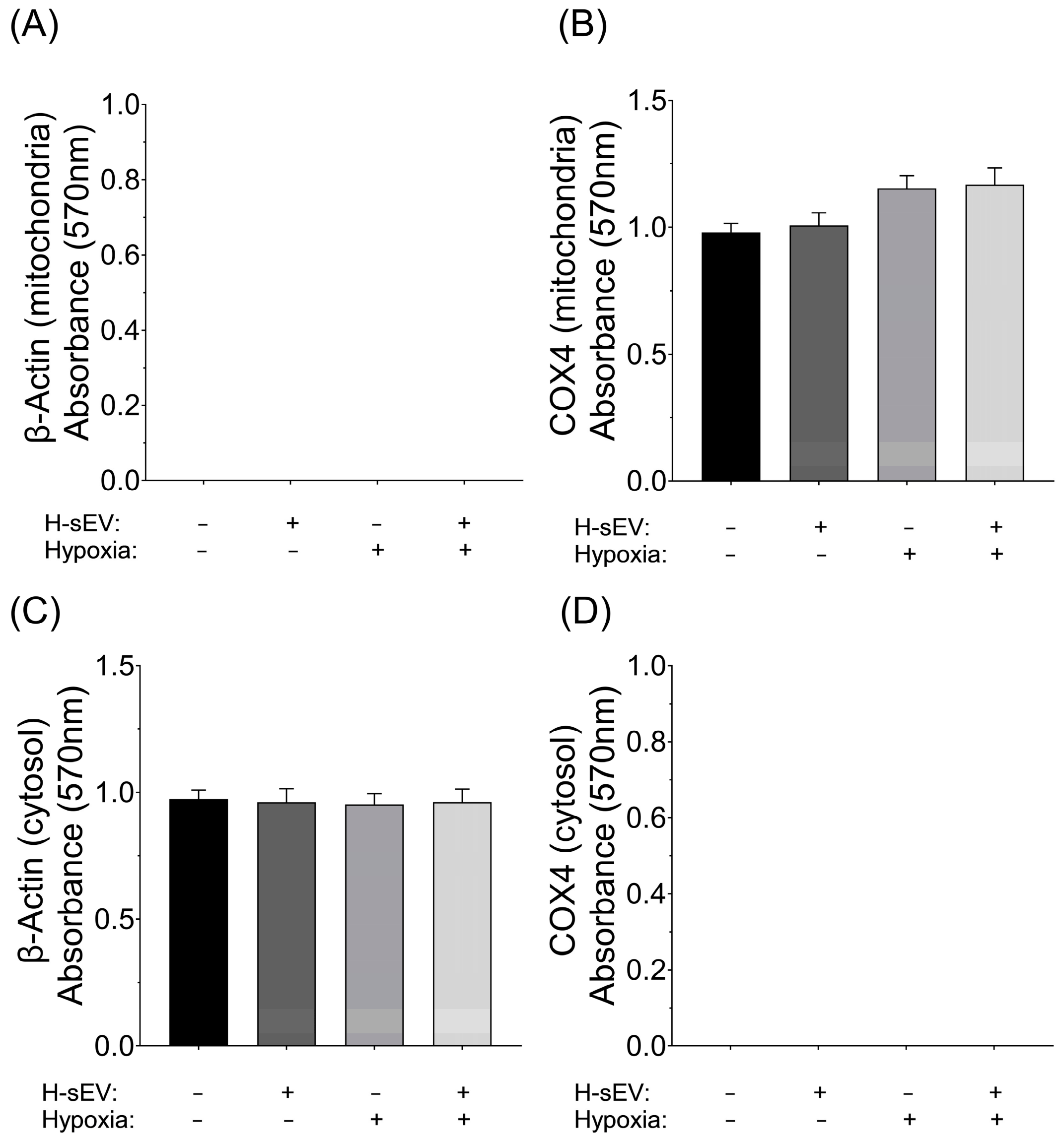

Fig.S1: Validation of the mitochondrial and cytosolic fractions by quantitative sandwich ELISA immunoassay. The mitochondrial fractions were validated by the absence of β-Actin (*A*) and the presence of COX4 (*B*). The cytosolic fractions were validated by the presence of β-Actin (C) and the absence of COX4 (*D*). Data was analyzed using one-way ANOVA multiple comparison analysis. Data from the validation assays is expressed as mean of raw values ± SEM (n = 4).
